# Proteome-wide survey of the autoimmune target repertoire in autoimmune polyendocrine syndrome type 1

**DOI:** 10.1038/srep20104

**Published:** 2016-02-01

**Authors:** Nils Landegren, Donald Sharon, Eva Freyhult, Åsa Hallgren, Daniel Eriksson, Per-Henrik Edqvist, Sophie Bensing, Jeanette Wahlberg, Lawrence M. Nelson, Jan Gustafsson, Eystein S. Husebye, Mark S. Anderson, Michael Snyder, Olle Kämpe

**Affiliations:** 1 Department of Medicine (Solna), Karolinska University Hospital, Karolinska Institutet, Sweden; 2 Science for Life Laboratory, Department of Medical Sciences, Uppsala University, Sweden; 3 Department of Genetics, Stanford University, California, USA; 4 Department of Molecular, Cellular, and Developmental Biology, Yale University, Connecticut, USA; 5 Department of Medical Sciences, Cancer Pharmacology and Computational Medicine, Uppsala University, Sweden; 6 Bioinformatics Infrastructure for Life Sciences, Sweden; 7 Department of Immunology, Genetics and Pathology, Uppsala University, Sweden and Science for Life Laboratory, Uppsala, Sweden; 8 Department of Molecular Medicine and Surgery, Karolinska Institutet, Stockholm, Sweden; 9 Department of Endocrinology and Department of Medical and Health Sciences and Department of Clinical and Experimental Medicine, Linköping University, Linköping, Sweden; 10 Integrative Reproductive Medicine Group, Intramural Research Program on Reproductive and Adult Endocrinology, National Institute of Child Health and Human Development, National Institutes of Health, Bethesda, MD 20892, USA; 11 Department of Women’s and Children’s Health, Uppsala University, Sweden; 12 Department of Clinical Science, University of Bergen, and Department of Medicine, Haukeland University Hospital, Bergen, Norway; 13 Diabetes Center, University of California San Francisco, USA

## Abstract

Autoimmune polyendocrine syndrome type 1 (APS1) is a monogenic disorder that features multiple autoimmune disease manifestations. It is caused by mutations in the *Autoimmune regulator (AIRE)* gene, which promote thymic display of thousands of peripheral tissue antigens in a process critical for establishing central immune tolerance. We here used proteome arrays to perform a comprehensive study of autoimmune targets in APS1. Interrogation of established autoantigens revealed highly reliable detection of autoantibodies, and by exploring the full panel of more than 9000 proteins we further identified MAGEB2 and PDILT as novel major autoantigens in APS1. Our proteome-wide assessment revealed a marked enrichment for tissue-specific immune targets, mirroring *AIRE*’s selectiveness for this category of genes. Our findings also suggest that only a very limited portion of the proteome becomes targeted by the immune system in APS1, which contrasts the broad defect of thymic presentation associated with *AIRE*-deficiency and raises novel questions what other factors are needed for break of tolerance.

Autoimmune responses can ultimately be defined at the molecular level by the specific interaction between T- or B-cell receptors and a distinct self-molecule. In tissue-specific autoimmune disorders the immune system typically target molecules that are exclusively expressed in the affected tissue and involve a combined cellular and humoral response with cognate specificities^1^,^2^,^3^. While the T-cell response is believed to directly effectuate tissue-destruction in many of these disorders, the B-cell response is best exploited for monitoring disease. Autoantibody markers have acquired a central role in research and clinical diagnosis of autoimmune disorders, providing means for secure diagnosis, stratifying patients into etiological subgroups and predicting future development of disease^4^. Autoantibody markers in current clinical use have been identified over decades, often through hypothesis driven efforts. Now developments in protein array technology have opened a novel avenue for explorative biomarker studies in autoimmune diseases and for capturing a higher degree of complexity in autoimmune responses^5^,^6^,^7^. Current-day protein arrays contain many thousands of full-length human proteins and enable autoantibody screens at the proteome-scale.

Autoimmune polyendocrine syndrome type 1 (APS1) is an autosomal recessive disorder that features multiple tissue-specific autoimmune manifestations (Mendelian Inheritance in Man #240300). APS1 is clinically defined by three hallmark components; chronic mucocutaneous *Candidiasis*, hypoparathyroidism and Addison’s disease, of which two are required for the diagnosis^8^. Most patients develop additional autoimmune disease manifestations in endocrine and non-endocrine organs, such as type 1 diabetes mellitus, pernicious anemia, vitiligo, alopecia and autoimmune ovarian insufficiency^8^,^9^. APS1 is caused by mutations in the *Autoimmune regulator (AIRE)* gene^10^,^11^, which has a key function in instructing the immune system to tolerate self. AIRE drives a promiscuous expression of peripheral tissue antigens in medullary thymic epithelial cells^12^. *AIRE*-driven antigen display ensures that naïve T-cells are exposed to tissue-specific molecules that otherwise would not be encountered in the thymus, and is therefore indispensible for the negative selection of autoreactive T-cells^12^,^13^. It has been estimated that about four thousand genes are presented under *AIRE*’s control in the thymus^14^,^15^,^16^. It has, however, remained elusive how many of these proteins, for which the filter of central tolerance fails, that become immune targets in patients with mutations in *AIRE*. Although several autoantigens have been defined in APS1 through focused investigations^17^,^18^,^19^,^20^,^21^,^22^, the range of self-molecules that become targeted by the immune system has not been studied in a comprehensive way. We here used human protein arrays to perform a proteome-wide survey of autoantibody targets in APS1, to enable a broad characterization of immune responses and to identity novel immune targets.

## Results

### Proteome array screening and survey of established autoantigens

**Figure 1:**
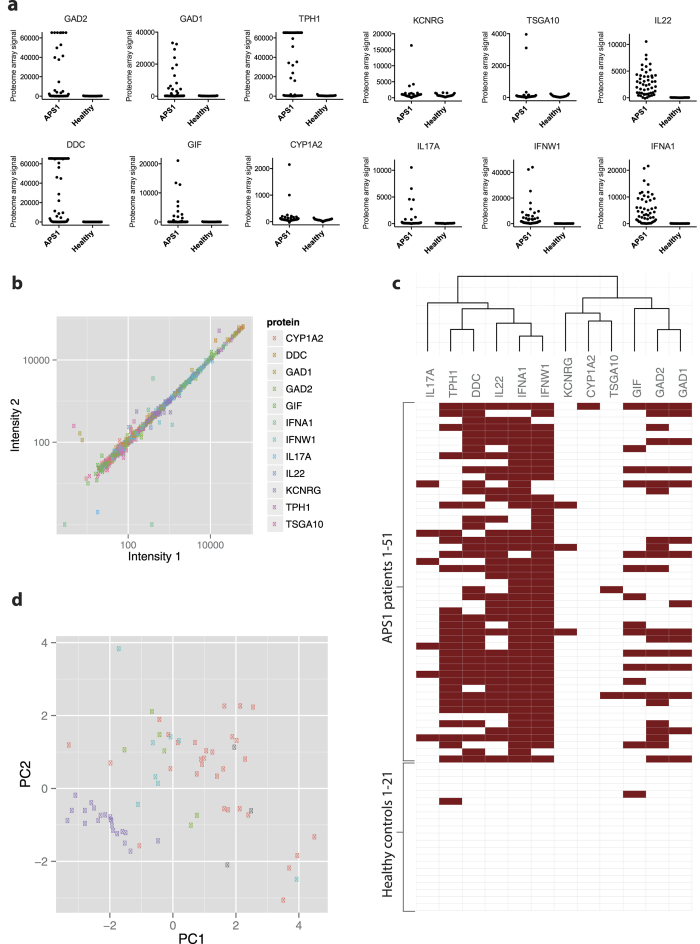
Survey of established autoantigens. Protein arrays containing over 9000 full-length human proteins were screened with sera from 51 patients with APS1 and 21 healthy controls for detection of IgG autoantibodies. A number of established autoantigens were present in the panel, including: glutamate decarboxylase 2 (GAD2), glutamate decarboxylase 1 (GAD1), tryptophan hydroxylase (TPH1), aromatic L-amino acid decarboxylase (DDC), gastric intrinsic factor (GIF), cytochrome P450, family 1, subfamily A, polypeptide 2 (CYP1A2), potassium channel regulator (KCNRG), testis specific, 10 (TSGA10), interleukin 22 (IL22), interleukin 17A (IL17A), interferon omega (IFNW1), interferon alpha 1 (IFNA1) and multiple other interferon alpha species. All established autoantigens displayed elevated patient-specific signals. Proteome array signals are represented as the average fluorescence signal intensities for protein duplicates on the array after subtraction of the background signal (**a**). All proteins were represented twice on the array, and protein duplicates of the established autoantigens showed excellent consistency in autoantibody signal levels (**b**). We assessed correlations between autoantibody responses against the different established autoantigens (**c**). Autoantibodies against the homologous antigens, IFNA1-IFNW1 and GAD2-GAD1, were strongly correlated. We also observed correlation between TPH1 and DDC that belong to distinct protein families but are coexpressed in serotonergic cells in the gut and nervous system. Autoantibody responses were clustered according to their Spearman correlations, using complete linkage hierarchical clustering. Distance = 1 – correlation. To evaluate effects of *AIRE* genotype on autoantibody expression we performed a principal component analysis using autoantibody data for the known autoantigens (**d**). The 21 healthy controls (purple) formed a distinct cluster separated from the patients with APS1, but patients homozygous for R257X (red), patients homozygous for c967−979del13 (green) and patients heterozygous for *AIRE* or with rare *AIRE* gene mutations (turquoise) were dispersed without tendency for internal clustering. *AIRE* genotype was not available for three of the patients (grey).

Patients with APS1 develop multiple autoimmune manifestations and harbor autoantibodies against a range of tissues. We used sera from 51 patients with APS1 and 21 healthy subjects to probe a human proteome arrays containing over 9000 full-length human proteins (ProtoArray®) for IgG autoantibodies. The great majority of array targets generated low signal intensities at around one thousandth of the saturation level for both patient and healthy control sera, and only a minimal fraction of the targets generated near saturated or saturated signal levels (see Supplementary Fig. S1). A number of established autoantigens were present in the panel, including: glutamate decarboxylase 2 (GAD2) (also known as GAD65), glutamate decarboxylase 1 (GAD1) (known as GAD67), tryptophan hydroxylase (TPH1), aromatic L-amino acid decarboxylase (DDC), gastric intrinsic factor (GIF), cytochrome P450, family 1, subfamily A, polypeptide 2 (CYP1A2), potassium channel regulator (KCNRG), testis specific 10 (TSGA10), interleukin 22 (IL22), interleukin 17A (IL17A), interferon omega (IFNW1), interferon alpha 1 (IFNA1) and multiple other interferon alpha species. These internal controls provided excellent means to evaluate the performance of autoantibody detection. All previously identified autoantigens were replicated in the screen, showing highly elevated signals specifically among patients (Fig. 1a). Array proteins were spotted twice in neighboring positions on the array, and duplicates for the established autoantigens exhibited excellent consistency in autoantibody signal levels (Fig. 1b). The established autoantibodies varied in frequency from 4–92% in the APS1 cohort (cutoff = average of the healthy subjects + 3SD), IFNW1 autoantibodies being the most frequently detected autoantibody, and were overall detected in the same range of frequency as previously reported^23^ (see Supplementary Table S1). To further evaluate the reliability of the proteome array data we validated the results for DDC autoantibodies in the 51 patients with APS1 using a radio-ligand binding assay, and the two independent methods showed excellent consistency (see Supplementary Figs S2–5). Collectively, our survey of known autoantigens in the proteome array screen revealed a highly reliable detection of autoantibodies.

Patients with APS1 develop complex combinations of autoimmune manifestations. We assessed correlations between the established autoantibodies to better understand how individual autoimmune responses were interlinked. Autoantibodies against the two closely related type 1 interferon specifies; IFNW1 and IFNA1, showed strong correlation (Spearman’s ρ = 0.79). Likewise, we observed correlations between autoantibodies against GAD2 and GAD1 (ρ = 0.70) that are both glutamic acid decarboxylases and co-expressed in pancreatic islet cells and gamma-aminobutyric acid (GABA) producing neurons. Interestingly, we also found correlation between TPH1 and DCC (ρ = 0.70), which belong to distinct enzyme families but are both involved in the monoamine synthesis and co-expressed in serotonin producing cells in the central nervous system and gut (Fig. 1c).

Around 100 different *AIRE*-gene mutations have been described in patients with APS1^24^. The most frequent mutations in APS1 are the Finnish mutation that causes a premature stop codon, R257X, and a 13-bp deletion in exon 8 that results a displaced reading frame, both disrupting first plant homeodomain (PHD1) zinc finger domain in the *AIRE* gene. With the exception of two described *AIRE* gene mutations associated with distinct non-APS1 autoimmunity syndromes with dominant inheritance^24^,^25^, no reliable genotype to phenotype relationships has been established for mutations in the *AIRE* gene^26^,^27^,^28^. To evaluate potential effects of *AIRE* genotype an autoimmune expression in APS1 we compared autoantibody responses against the known antigens between patients with different *AIRE* genotypes. We first performed a principal component analysis using autoantibody data for the known autoantigens and investigated whether patients grouped according to their *AIRE* genotypes. The 21 healthy controls formed a distinct cluster separated from the patients with APS1, but patients homozygous for R257X, patients homozygous for c967−979del13 and patients heterozygous for *AIRE* or with rare *AIRE* gene mutations were dispersed without tendency for internal clustering (Fig. 1d). To further assess whether there was relativeness between patients with shared *AIRE* mutations we clustered the patients with APS1 and the healthy controls in a dendrogram according to their Spearman correlations for the known autoantibodies (see Supplementary Fig. S6). The healthy controls grouped together while patients with APS1 did not show appreciable tendency for clustering according to type of *AIRE* mutation. *AIRE* genotype thereby did not appear to be an important determinant of autoantibody expression.

Cytokine autoantibodies in APS1 have gained high interest because of their link with chronic mucocutaneous *Candidiasis* and also because of their high prevalence in patients with APS1 that allows for sensitive blood marker based diagnosis^29^,^30^,^31^. We made a focused investigation of 49 interferons, interleukins and other closely related cytokines present on the array, to enable a detailed profiling of cytokine autoantibodies in our patients. High autoantibody signals were specifically seen for the previously reported immune targets, including multiple alpha interferon specifies, IFNW1, IL22 and IL17A (IL17F was not present in the array panel) (see Supplementary Fig. S7). Autoantibodies against the alpha interferons were strongly correlated. To discern whether the observed correlations represented biological variation in autoantibody responses against the different alpha interferon species or could be fully explained by technical variation we compared against the correlation between IFNA1 and IFNA13. The genes for IFNA1 and IFNA13 encode an identical protein product, and the correlation between INFA1 and IFNA13 could therefore be used as a reference point representing the level of technical variation in cytokine autoantibody detection. Although INFA1 and IFNA13 were strongly correlated (ρ = 0.96), suggesting the technical variation was low, there were several interferons that correlated at or near the same level as between IFNA1 and IFNA13. Autoantibody responses between the different alpha interferon species thereby appeared to be closely associated, consistent with a scenario of a shared autoepitopes and extensive autoantibody cross-reactivity. We also noted that IFNA4 produced the by far strongest autoantibody signal of the interferons. Although the signal intensity is expected to depend in part on factors specific for the detection method, the observation suggested that IFNA4 could be a dominating interferon target and possibly better suited than other interferons for diagnostic autoantibody assays.

### Proteome-wide survey identifies PDILT and MAGEB2 as novel immune targets

**Figure 2:**
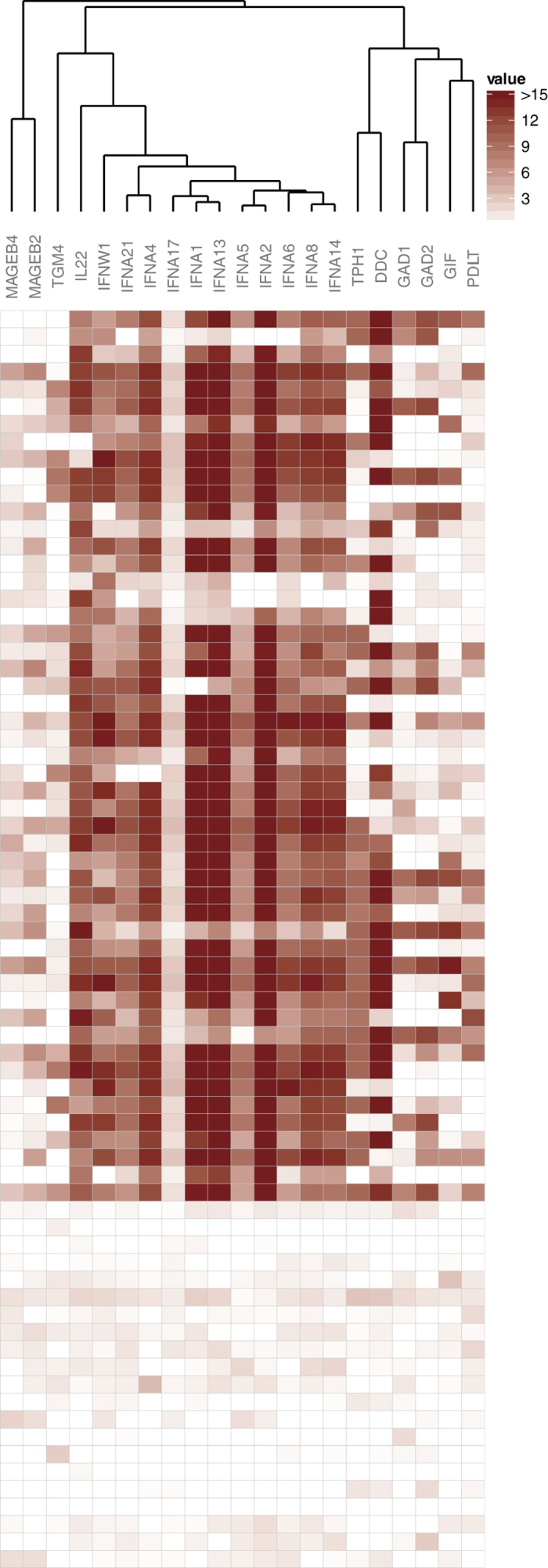
Major immune targets in the proteome array screen. We assessed the full panel of more than 9000 human proteins to identify the major immune targets in the patients with APS1. As a first filtration we excluded all targets that failed to reach a signal intensity of more than 5000 in any of the investigated sera or more than 2000 in the negative sample (array probed with only detection reagent and no serum). To select for patient-specific signals we then compared the frequency of positive individuals between patients with APS1 and the healthy control group using Fisher´s exact test (cutoff = average of the healthy + 3SD). Among the top signals, a number of printing contamination artifacts were identified and excluded. Our annotated set of major immune targets in the proteome array screen then contained eleven type one interferon specifies, another six known autoantigens (IL22, TPH1, DDC, GAD1, GAD2 and GIF), transglutaminase 4 (TGM4)^32^, and three novel candidate autoantigens - protein disulfide isomerase-like testis expressed (PDILT), melanoma antigen family B 2 (MAGEB2) and MAGEB4. Autoantibody signals are clustered according to their Spearman’s correlations using complete linkage hierarchical clustering. Distance = 1 – correlation.

**Figure 3:**
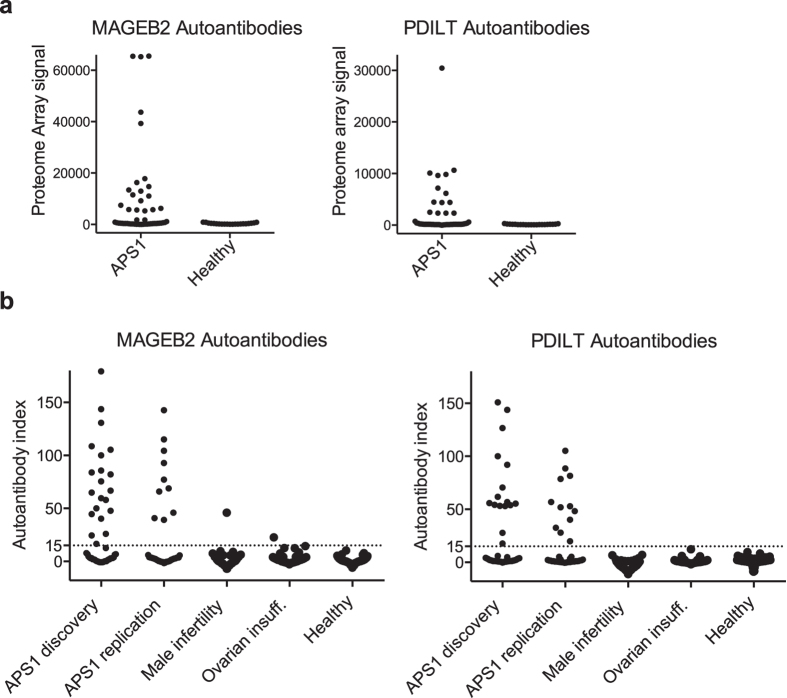
Identification of MAGEB2 and PDILT as major autoantigens in APS1. MAGEB2 and PDILT were identified as major immune targets in the proteome array screen. MAGEB2 autoantibodies were detected in 21 out of 51 (41%) patients with APS1 and PDILT autoantibodies in 19 of 51 (37%) patients with APS1, while absent in controls (cutoff = average of the healthy + 3 SD) (**a**). Radio-ligand binding assays were used to validate MAGEB2 and PDILT autoantibodies in the discovery cohort, in a replication cohort of 42 patients with APS1 and in control cohorts that included females with premature ovarian insufficiency (n = 114), males with idiopathic infertility (n = 76) and healthy subjects (n = 91) (**b**). In total MAGEB2 autoantibodies were detected in 31 out of 92 (34%) patients with APS1, in a male with idiopathic infertility and a female with premature ovarian insufficiency, while absent in all healthy controls. PDILT autoantibodies were detected in 28 out of 93 (30%) patients with APS1 and were absent in all healthy and all disease controls (cutoff = index-value 15).

Our review of known autoantigens demonstrated highly reliable detection of autoantibodies, and we next interrogated the full panel to identify novel immune targets. As an initial filtration we excluded all targets that failed to reach a signal intensity of more than 5000 in any of the investigated sera or more than 2000 in the negative sample (array probed with only detection reagent and no serum), which left us with a set of 429 targets for further investigation. To select for patient specific signals we introduced cutoff values for all targets at three standard deviations above the average of the healthy control group and compared the frequency of positive individuals between the patients with APS1 and the healthy control group. The 35 targets most strongly associated with the APS1 patient group, with unadjusted p-values ranging from 8 × 10^−19^ to 0.05 (Fisher´s exact test), were selected for further scrutiny. In this selection we found several targets that were located nearby and strongly correlated with known autoantigens on the array, suggesting these signals had appeared as result of printing contaminations (see Supplementary Figs S8–9). To identify and exclude printing contamination artifacts among our selected candidates we assessed the correlation between the 35 top targets and excluded those showing strong and unexpected correlations with nearby located targets. Fourteen artifacts could be identified and excluded in this process (see Supplementary Fig. S9). Our annotated set of top signals, representing the major immune targets in the proteome array screen, then contained eleven type one interferon species (IFNW1, IFNA21, IFNA4, IFNA17, IFNA1, IFNA13, IFNA5, IFNA2, IFNA6, IFNA8 and IFNA14), another six previously known autoantigens (IL22, TPH1, DDC, GAD1, GAD2 and GIF), transglutaminase 4 (TGM4)^32^, and three novel candidate autoantigens - melanoma antigen family B 2 (MAGEB2 and MAGEB4) and protein disulfide isomerase-like testis expressed (PDILT) (Fig. 2). MAGEB2 and MAGEB4 are members of the same protein family and show 66% protein sequence identity BLAST comparison (NP_002358.1 vs NP_002355.2). The autoantibody signals for MAGEB2 and MAGEB4 were correlated (ρ = 0.65), suggesting they were both targets of a shared cross-reactive response. MAGEB2 autoantibodies were detected in a greater number of patients and showed consistently higher signal intensities than MAGEB4, suggesting MAGEB2 was the dominating autoantigen. Autoantibodies against MAGEB2 were detected in 21 out of 51 patients with APS1 and were absent in all controls. PDILT autoantibodies were present in 19 of 51 patients with APS1 while absent in controls (Fig. 3a).

### Validation of MAGEB2 and PDILT as major gonadal autoantigens

Our proteome array data suggested MAGEB2 and PDILT were major immune targets in APS1. To confirm the novel candidates with an independent method we developed radio-ligand binding assays for MAGEB2 and PDILT and assessed an extended APS1 cohort and a broad clinical control material. Previous studies suggest MAGEB2 and PDILT are specifically expressed in male gonadal cells^33^,^34^,^35^, and studies on murine MAGEB4^36^ further indicate MAGEB2 may also be expressed in oocytes. MAGEB2 and PDILT autoantibodies were present in both male and female patients with APS1 at comparable frequencies. We therefore included control subjects with idiopathic male infertility^37^ (n = 76) and premature ovarian insufficiency^38^ (n = 114), together with healthy controls (n = 91). Radio-labeled human MAGEB2 and PDILT protein was produced in a cell-free eukaryotic expression system and was subjected to immunoprecipitation with APS1 patient and control sera. The radio-ligand binding assays confirmed presence of MAGEB2 and PDILT autoantibodies in the discovery cohort (Fig. 3b), and showed excellent consistency with the proteome array results in classifying patients as reactive or non-reactive (see Supplementary Figs S10 and S12). We assessed a replication APS1 cohort, where MAGEB2 autoantibodies were detected in 9 out of 41 and PDILT autoantibodies in 12 out of 42 patients. In total MAGEB2 autoantibodies were detected in 31 out of 92 (34%) patients with APS1, in a single male with idiopathic infertility, just above cutoff in a female with premature ovarian insufficiency and were absent in all healthy controls. PDILT autoantibodies were detected in 28 out of 93 (30%) patients with APS1 and were absent in all healthy and all other controls. MAGEB2 and PDILT autoantibodies thereby appeared to be highly specific for APS1.

**Figure 4:**
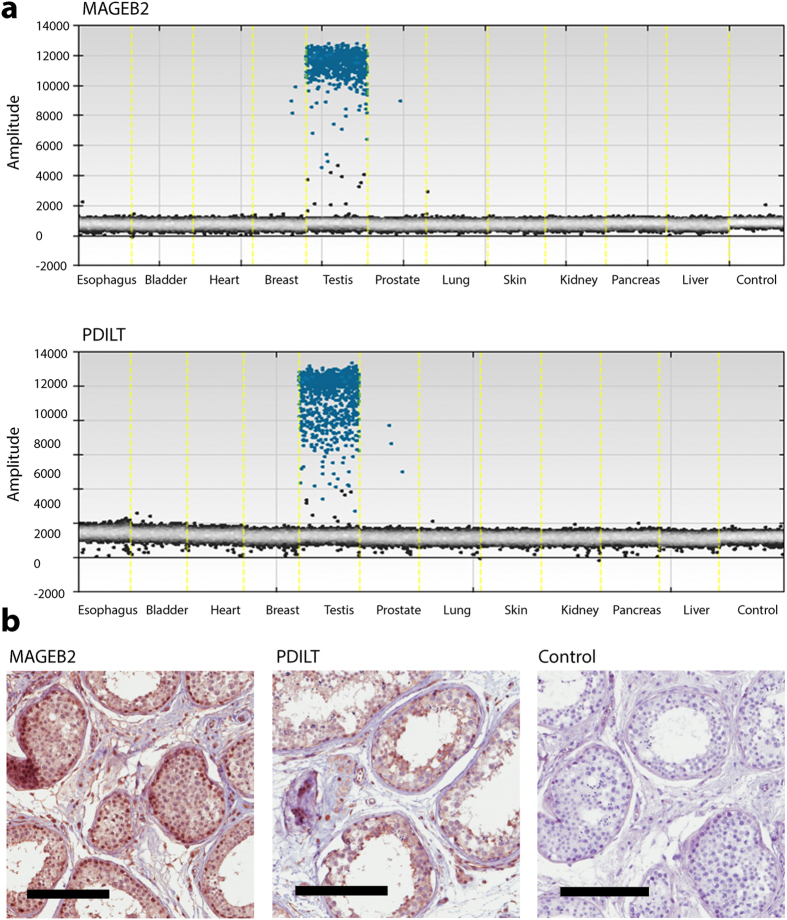
MAGEB2 and PDILT are specifically expressed in gonadal germ cells. The mRNA distribution of MAGEB2 and PDILT was investigated across eleven human tissue samples by digital droplet PCR. MAGEB2 and PDILT transcripts were specifically detected in the testicular tissue (**a**). To investigate the cellular distribution of MAGEB2 and PDILT within the testis we performed immunohistochemistry on human testicular tissue (**b**). The MAGEB2-antiserum stained germ cells of all differentiation stages. Unspecific nuclear positivity was also observed. The PDILT-antiserum stained predominantly for a subset of germ cells located near the lumen of the seminiferous tubule. A negative control is included where the primary antibody was omitted from the staining procedure.

We validated the tissue-distribution of MAGEB2 and PDILT mRNA across a panel of eleven human tissues using digital droplet PCR. MAGEB2 and PDILT transcripts were detected at high levels in testicular tissue and were absent in the non-gonadal tissues (Fig. 4a and Supplemental Fig. S12). To define the cellular distribution of MAGEB2 and PDILT within the testis we performed immunostaining on human testicular tissue. The MAGEB2 antiserum labeled germ cells across all differentiation stages. The PDILT antiserum stained predominantly for a subset of germ cells located near the lumen of the seminiferous tubule corresponding to cells of late differentiation stages in spermatogenesis, consistent with previous reports^34^ (Fig. 4b). Collectively, our findings suggested MAGEB2 and PDILT are major gonadal autoantigens in APS1.

## Discussion

APS1 features multi-organ autoimmunity and autoantibody responses against a range of tissues. Although several immune targets have been defined in separate studies, the autoimmune expression of APS1 has not previously been captured in a comprehensive way. In this study we assessed the B-cell response against a panel of over 9000 human proteins to enable detailed profiling of known autoantigens and to identify novel immune targets in APS1. Review of multiple established autoantigens revealed highly robust autoantibody detection, and by exploring the full array panel we further identified two novel autoantigens, MAGEB2 and PDILT, that could be confirmed by independent methods and in a replication cohort.

Studies in APS1 and its animal model have shed light on the process whereby the immune system learns self tolerance^12^. It has, however, remained incompletely understood how a defect of central tolerance in *AIRE*-deficiency is translated into autoimmune responses, as comprehensive studies of autoimmune targets have not been previously undertaken in patients with APS1 or *Aire*-deficient mice. Our broad characterization of B-cell targets provides a novel means to appreciate these events.

One important observation that can be made is that only a very limited portion of the proteome appears to become targeted by autoantibodies in patients with APS1, as compared with the broad defect in thymic antigen presentation associated with *AIRE*-deficiency. Studies in *Aire* knock out mice have allowed detailed characterization of *AIRE* controlled gene expression - first using micro arrays and PCR and recently with increased precision by mRNA sequencing - and suggest around four thousand genes are positively controlled by *Aire* in medullary thymic epithelial cells^12^,^14^,^15^,^16^. We interrogated the immune response against around one third of the annotated human proteins^39^ and found only around 20 antigens that were frequent immune targets in the patients with APS1. This leaves a vast discrepancy between the number of genes that are presented under control of *AIRE* and the number of molecules that become immune targets in APS1. It is important here to consider the possibility that some autoantigens in the panel could have failed to generate signals of technical reasons and therefore were missed in our analyses. Indeed autoantibody detection can depend on factors such as protein folding, posttranslational modification and immobilization of the antigen. However, our extensive review of previously established autoantigens, replicating all known targets in the panel, indicated a highly sensitive and reliable detection of autoantibodies. The great difference in number of *AIRE* controlled genes and number of detected autoantigens thereby did not appear to represent a technical bias but to reflect biology.

Several explanations must be considered why the autoimmune response is, as it appears, orders of magnitude more limited than could be expected from the defect in *AIRE*-dependent antigen presentation. First it should be recognized that antigen recognition is MHC-dependent, and that only a subset of self-antigens are expected to possess the necessary requirements for being presented on MHC efficiently and to render sturdy immune responses. The potential impacts of B-cell tolerance and peripheral tolerance mechanisms must also be considered here, which may provide additional filters for the development of autoimmune responses. Recent studies in patients with APS1 have also shed light on the role of peripheral antigen presentation in autoimmune manifestation^32^. It was found that autoantibodies against a prostate-specific enzyme only developed in male patients and first after the age of pubertal prostate maturation, indicating peripheral antigen expression was a necessary for the development of the autoimmune response. This rare example of a sex dimorphic autoantigen suggests *AIRE*-dependent genes must also be expressed at sufficient level in the periphery in order to give rise to autoantibody responses.

Another key observation that can be made from our survey of autoantibody specificities is that autoimmunity in APS1 preferentially targets molecules with restricted tissue expression profiles. This conforms to previous experience, and stands out with increased clarity here with the overview perspective from a proteome-wide screen. In our selection of major immune targets more than half were annotated as being tissue enriched and remaining as either being tissue enhanced, group enriched or not detected, according to mRNA sequencing data from the *Human Protein Atlas*^40^ (Table S2). This should be compared to the whole genome, where only twelve percent of the genes are annotated as being tissue enriched^40^. Notable, while almost half of the genes in the genome are annotated as being expressed in all tissues we found no immune targets in our proteome array screen that belonged to this category. This marked enrichment for tissue-specific immune targets goes well in hand with the known disease biology of *AIRE*-deficiency and the notion that *AIRE* is responsible for specifically compensating the absence of tissue-specific genes in the thymus^12^. Ubiquitously expressed proteins are expected to be presented in the thymus and render toleration equally in patients with APS1 as in healthy individuals.

We also investigated whether the APS1 autoantigens shared functional characteristics parts from being tissue-specific, and searched for over represented gene ontology terms among the major immune targets in the screen. The most frequently shared ontology term related to *amino acid and derivate metabolism* and *cell-cell signaling.* Also *neurotransmitter biosynthesis* and *vitamin binding* were enriched terms among the immune targets (see Supplementary Table S3).

We identify MAGEB2 and PDILT as novel, major autoantigens in APS1. Interestingly, both MAGEB2 and PDILT are specifically expressed in gonadal germ cells. MAGEB2 is a member of a gene family clustered on the *dosage-sensitive sex reversal* region of the X-chromosome, and is expressed in testis tissue and also a variety of tumors^35^. PDILT is a testis-specific protein that shows homology with disulfide isomerases^33^,^34^,^41^. PDILT functions in a germ cell-specific chaperone complex that is necessary for male fertility^41^. Notably, despite apparent testis-specific expression, MAGEB2 and PDILT autoantibodies were present in both male and female patients with APS1. This observation, together with experience from a prostate autoantigen in APS1^32^, raises the question whether MAGEB2 and PDILT may also be expressed in female tissues. MAGEB genes are indeed expressed in female germ cells and appear to have important roles in female gametogenesis^36^. It is believed that many genes with apparent testis-specific expression are also expressed in the female germ cells and that oocyte expression is easily overlooked in analyses of gross ovarian tissue^40^.

The gonadal-specific expression of MAGEB2 and PDILT raises the questions whether these antigens may have a role in infertility in APS1. Infertility is common in both males and females with APS1^8^. Female infertility is in most cases explained by premature ovarian insufficiency and autoimmunity against ovarian steroid producing cells. Prostate autoimmunity was recently identified as a major manifestation of APS1 with possible role in the development of male infertility^32^. The identification of MAGEB2 and PDILT reveals the gonadal germ cells as major immune targets in APS1, and discloses yet another autoimmune component that potentially could contribute to infertility in male and female patients with APS1.

So far most studies attempting to use protein array technology to evaluate autoantibody reactivity have been carried out in disorders without an established autoimmune etiology, where known autoantigens have not been available as internal controls and where it also has proved challenging to identify novel targets that reliably separate cases from controls. APS1 is a well-characterized autoimmune disorder with several defined autoantigens, and our screens with APS1 sera allowed for a more stringent evaluation of the proteome array performance than has been possible before. The many known immune targets present in the panel revealed highly reliable detection of autoantibodies, but also exposed erroneous artifacts from printing contaminations. Artifacts of this kind could easily have gone unnoticed in studies of sera without internal controls in the form of previously defined immune targets. While printing contamination artifacts on a proteome array can be handled if identified, such effects would be detrimental for customized miniature arrays where antigens of interest are typically spotted in neighboring positions. The strategy we used of assessing correlations between signals on the array can be an efficient way of identifying artifacts due to printing contaminations in large data sets. In design of focused arrays it may be well worthwhile to include control spots in-between targets of interest, to be able to control for and isolate the effects of printing contaminations. Overall our screen with APS1 sera showed results that are very encouraging for future proteome arrays studies in other autoimmune disorders. One attractive application would for example be to explore acquired immunodeficiencies, where previous work has linked some of these disorders with a pathogenesis involving neutralizing autoantibodies against distinct cytokine species^42^,^43^. Our survey of cytokine autoantibodies in APS1 reliably identified the expected targets, suggesting these arrays would be well suited for this purpose. The proteome array results also open up for novel ways to assess clinical markers in APS1 more efficiently. Focused arrays can be designed to include most of the known autoantigens in APS1, and would allow for high throughput and highly informative autoantibody screens in patients with APS1. Antigen panels of this sort could also be of great clinical interest for screening patients with other autoimmune disorders, such as type 1 diabetes, autoimmune thyroiditis and Addison’s disease in the general population, where the assessment of well-defined autoantibody markers may be useful for identifying occult autoimmune diseases and predicting future development of disease.

## Methods

### Patient samples

Serum samples were collected from Finnish, Norwegian and Swedish patients with APS1. All patients met the clinical diagnostic criteria for APS1, requiring at least two of the hallmark components; chronic mucocutaneous *Candidiasis*, hypopararthyroidism and adrenal failure, and most patients had also been genotyped and found with mutations in the *AIRE* gene. Hallmark components were defined as the following; chronic mucocutaneous *Candidiasis* - *Candida* infection of the oral mucosa, skin or nails for a period of more than three months, hypoparathyroidism: plasma calcium concentration below 2.15 and elevated plasma phosphate concentration in combination with normal or low parathormone concentration and normal renal function, adrenal failure: sub-normal serum cortisol in combination with elevated plasma adrenocorticotropic hormone (ACTH) concentration or deficient response to synthetic ACTH stimulation test (failure to reach 550 nmol/L in 30 or 60 min). Most patients had also been diagnosed with additional disease components, including; malabsorption, type 1 diabetes mellitus, hypogonadism, pernicious anemia, alopecia and vitiligo. Females with premature ovarian insufficiency not related to APS1^44^, males with idiopathic infertility not related to APS1^37^ and healthy blood donors were included as controls. The project was conducted in accordance with the Helisinki declaration and was approved by ethical boards in Helsinki, Bergen and Uppsala (project code: Ups 02-415).

All patients had given their informed consent for participation.

### Protein array screening

Probing and scanning of the protein arrays (ProtoArray® v5.0 PAH0525020, Life Technology) was conducted according to Invitrogen’s protocol for *Immune Response BioMarker Profiling*, using the recommended detection reagent (Alexa Fluor® 647 Goat Anti-Human IgG A21445, Invitrogen) and blocking buffer (Blocking Buffer Kit PA055, Invitrogen). The arrays were probed with sera at a dilution of 1:2000. Arrays were scanned using a GenePix 4000B microarray scanner, and the GenePix® Pro microarray (v6.1) software was used for alignment and data acquisition.

### Statistical analyses

Statistical analyses of proteome array data were performed on log-transformed intensities. Fisher’s exact test was used for ranking protein array targets that differed between the patient with APS1 and healthy control group. A signal was considered positive if the intensity value was above the average of the healthy + 3SD.

### PDILT and MAGEB2 radio-ligand binding assays

Autoantibodies against PDILT and MAGEB2 were measured using radio-ligand binding assays. Human cDNA-clones for PDILT (SC306958, Origene, accession no. NM_174924) and MAGEB2 (SC122625, Origene, accession no. NM_002364) were cloned into a pTNT expression vector (L5610, Promega), and were transcribed and translated *in vitro* in presence of ^35^S Methionine (Promega TNT Systems). Immunoprecipitation was conducted in 96-well filtration plates (Millipore). A positive standard, an APS1 patient serum with known reactivity, and a negative standard, 4% BSA, were included to each plate. 30,000 CPM of radio-labeled protein and 2.5 μl of serum sample were added to each well. All sera were analyzed twice. Serum antibodies were immobilized to protein A Sepharose (nProtein A Sepharose^TM^ 4 Fast Flow, GE Health Care) during over-night incubation. Radioactivity was measured in a micro-beta counter (1450 Microbeta Trilux, Wallac). Autoantibody index values were calculated according to the following: (sample value/negative standard value)/(positive standard value/negative standard value) × 100.

### Digital droplet PCR expression assay

Total RNA from Origene was converted to cDNA using Life Technologies’ Double Stranded cDNA Synthesis Kit and the protocol with random hexamers (N8080127, Life Technologies) for the first strand synthesis. Bio-Rad’s ddPCR supermix for probes (186-3040, BioRad) was used and droplet generation was conducted according to BioRad’s standard protocol with TagMan probes to PDILT and MAGEB2 and β2M as a control (1206926, 4351372 and 4448489, Life Technologies). Samples were run on a Quantalife ddPCR machine and Quantasoft software was used to collect and analyze data.

### Immunohistochemistry

Staining for MAGEB2 and PDILT were performed on formalin fixed paraffin embedded human testicular tissue using the Autostainer 480 (Thermo Fisher Scientific). Tissue slides were first incubated with Ultra V block (TA-125-UB, Thermo Fisher Scientific, Inc.) for 5 minutes, and thereafter incubated with either anti-MAGEB2 (49-718, ProSci) at 1:1750 dilution or anti-PDILT (HPA041923, Sigma) at 1:1000 dilution for 30 minutes. The slides were then incubated with labeled horseradish peroxidase-polymer for 30 minutes, followed by 3,3′-Diaminobenzidine (DAB) solution for 2 × 5 minutes. Slides were counterstained in Mayers hematoxylin (01820, Histolab) for 5 minutes using the Autostainer XL (Leica), and then rinsed in lithium carbonate water (diluted 1:5 from saturated solution) for 1 minute. The slides were dehydrated in graded ethanol and lastly coverlipped (PERTEX, Histolab) using an automated glass coverslipper (CV5030, Leica). The slides were scanned using the automated scanning system Aperio XT (Aperio Technologies).

### Gene ontology enrichment analysis

To find statistically overrepresented gene ontology terms (GOa-human database) assigned to the established autoantigens present on the array, we used the online GOstat software^45^. To avoid interferons from outcompeting other genes in the GO term enrichment analysis completely, the interferons were represented by IFNA13 and IFNW1 only.

## Additional Information

**How to cite this article**: Landegren, N. *et al.* Proteome-wide survey of the autoimmune target repertoire in autoimmune polyendocrine syndrome type 1. *Sci. Rep.* **6**, 20104; doi: 10.1038/srep20104 (2016).

## Supplementary Material

Supplementary Dataset 1
