# Supplementary material for: Proteome-wide survey of the autoimmune target repertoire in autoimmune polyendocrine syndrome type 1
Source: Sci Rep. 2016 Feb 1;6:20104. doi: 10.1038/srep20104 (PMC4735587; doi:10.1038/srep20104)
Supplement: Supplementary Dataset 1 [file srep20104-s1.pdf]

Supplementary material

Proteome-wide survey of the autoimmune target repertoire in autoimmune polyendocrine syndrome type 1

*Nils Landegren[1,2], Donald Sharon[3,4], Eva Freyhult[2,5,6,], Åsa Hallgren[1,2], Daniel Eriksson[1,2], Per-Henrik Edqvist[7], Sophie Bensing[8], Jeanette Wahlberg[9], Lawrence M. Nelson[10], Jan Gustafsson[11], Eystein S Husebye[12], Mark S Anderson[13], Michael Snyder[3], Olle Kämpe[1,2]

Nils Landegren and Donald Sharon contributed equally to the work

Affiliations

[1]Department of Medicine (Solna), Karolinska University Hospital, Karolinska Institutet, Sweden

[2]Science for Life Laboratory, Department of Medical Sciences, Uppsala University, Sweden

[3]Department of Genetics, Stanford University, California, USA

[4]Department of Molecular, Cellular, and Developmental Biology, Yale University, Connecticut, USA

[5]Department of Medical Sciences, Cancer Pharmacology and Computational Medicine, Uppsala University

[6]Bioinformatics Infrastructure for Life Sciences

[7]Department of Immunology, Genetics and Pathology, Uppsala University, Sweden and Science for Life Laboratory

[8] Department of Molecular Medicine and Surgery, Karolinska Institutet, Stockholm, Sweden

[9]Department of Endocrinology and Department of Medical and Health Sciences and Department of Clinical and Experimental Medicine, Linköping University, Linköping, Sweden

[10]Integrative Reproductive Medicine Group, Intramural Research Program on Reproductive and Adult Endocrinology, National Institute of Child Health and Human Development, National Institutes of Health, Bethesda, MD 20892, USA.

[11]Department of Women's and Children's Health*, Uppsala University, Sweden*

[12]Department of Clinical Science, University of Bergen, and Department of Medicine, Haukeland University Hospital, Bergen, Norway

[13]Diabetes Center, University of California San Francisco, USA

*Correspondence should be addressed to

Nils Landegren

nils.landegren@ki.se

Department of Medicine Solna, Karolinska Institute

Experimental endocrinology

Center for Molecular Medicine, L8:01, Karolinska University Hospital

SE-171 76 Stockholm

nils.landegren@ki.se

phone: +46851779158

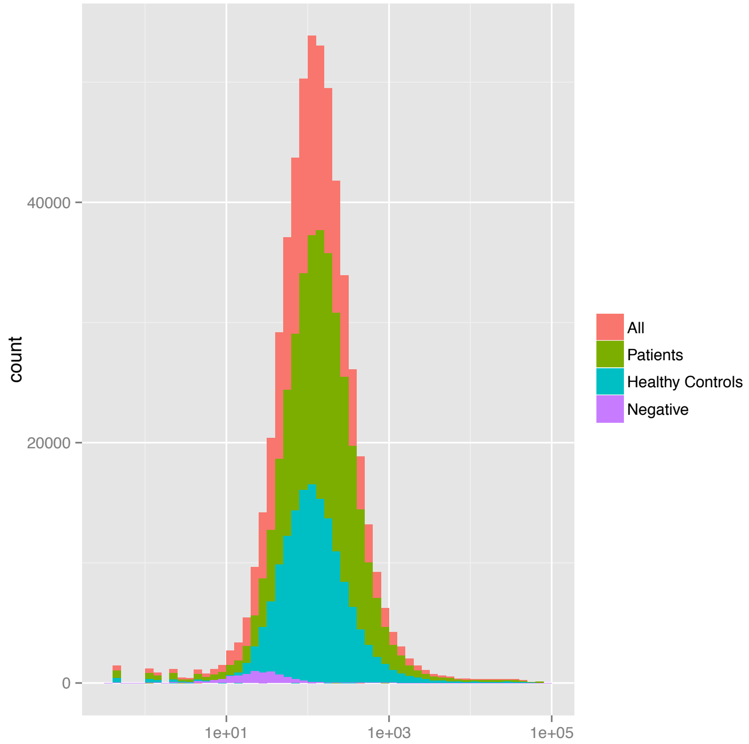

Fig. S1. Distribution of log-intensities for arrays probed with patient sera, healthy control sera or without serum sample (Negative).

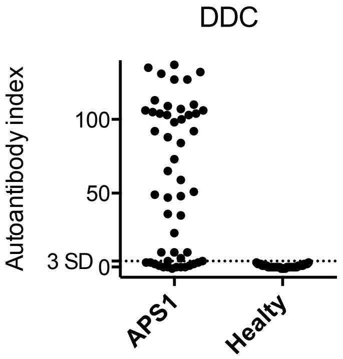

Fig. S2. The proteome array results for DDC autoantibody detection were validated using a radio-ligand binding assay (RLBA) in the 51 patients from the APS1 discovery cohort and in 39 healthy controls. The upper limit of the normal range was defined as three standard deviations above the average of the healthy controls.

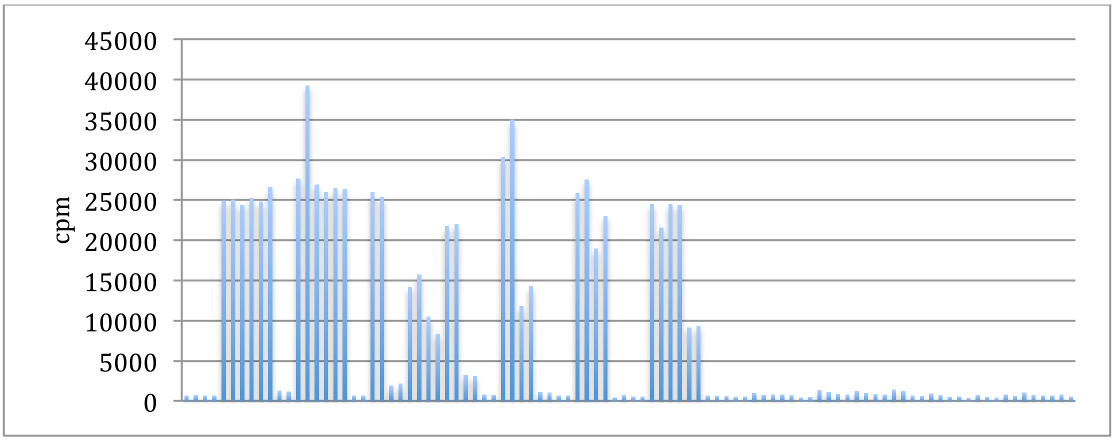

Fig. S3. Representative raw data from the DDC autoantibody radio-ligand binding assay. All patient and control samples were analyzed in duplicate. Negative control (bar 1-4), positive control (bar 5-8), patients with APS1 (bar 9-68) and healthy control subjects (bar 69-96). Counts per minute (CPM).

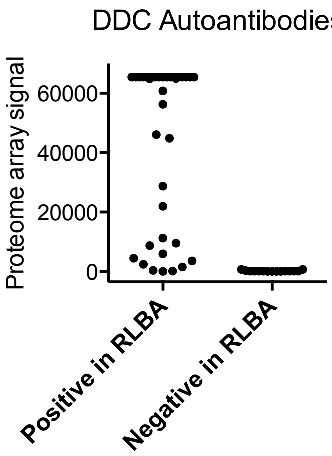

Fig. S4. The proteome array results for DDC were compared against the results from the radio-ligand binding assay (RLBA) for the 51 patients in the APS1 discovery cohort. Patients who were positive for DDC autoantibodies in the RLBA are grouped to the left (cut-off = average of the healthy + 3SD). Proteome array signals are

represented as the average fluorescence signal intensities for protein duplicates on the array after subtraction of the background signal.

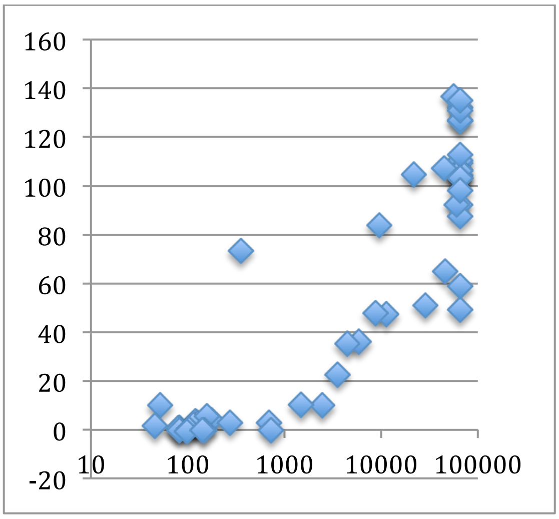

Fig. S5. The proteome array results for DDC were compared against the results from the radio-ligand binding assay (RLBA) for the 51 patients in the APS1 discovery cohort. Proteome array signal results are shown on the x-axis and RLBA results on the y-axis. Proteome array signals are represented as the average fluorescence signal intensities for protein duplicates on the array after subtraction of the background signal. RLBA results are shown as autoantibody index values.

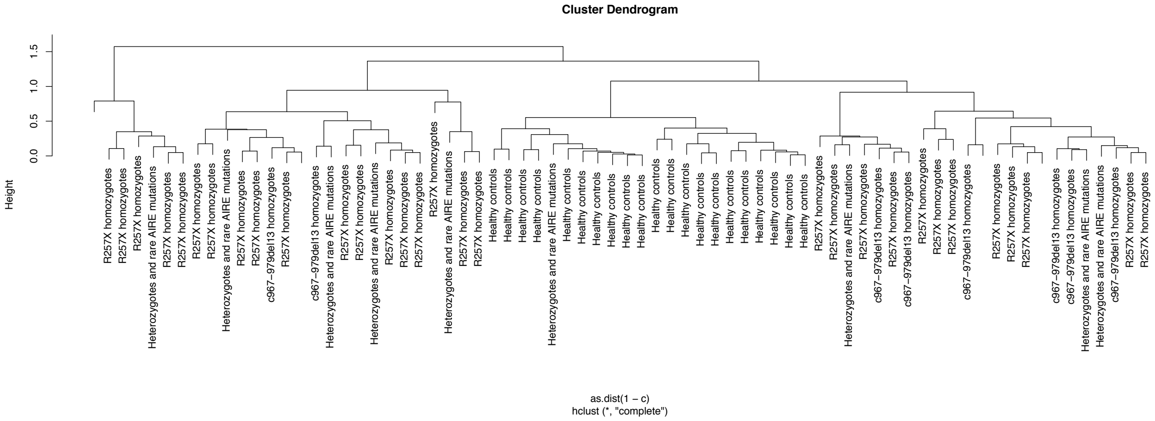

Fig. S6. Patients with APS1 and healthy controls were clustered based on Spearman correlations for the known autoantigens; glutamate decarboxylase 2 (GAD2), glutamate decarboxylase 1 (GAD1), tryptophan hydroxylase (TPH1), aromatic L-amino acid decarboxylase (DDC), gastric intrinsic factor (GIF), cytochrome P450, family 1, subfamily A, polypeptide 2 (CYP1A2), potassium channel regulator (KCNRG), testis specific 10 (TSGA10), interleukin 22 (IL22), interleukin 17A (IL17A), interferon omega (IFNW1) and interferon alpha 1 (IFNA1). Distance = 1 – correlation.

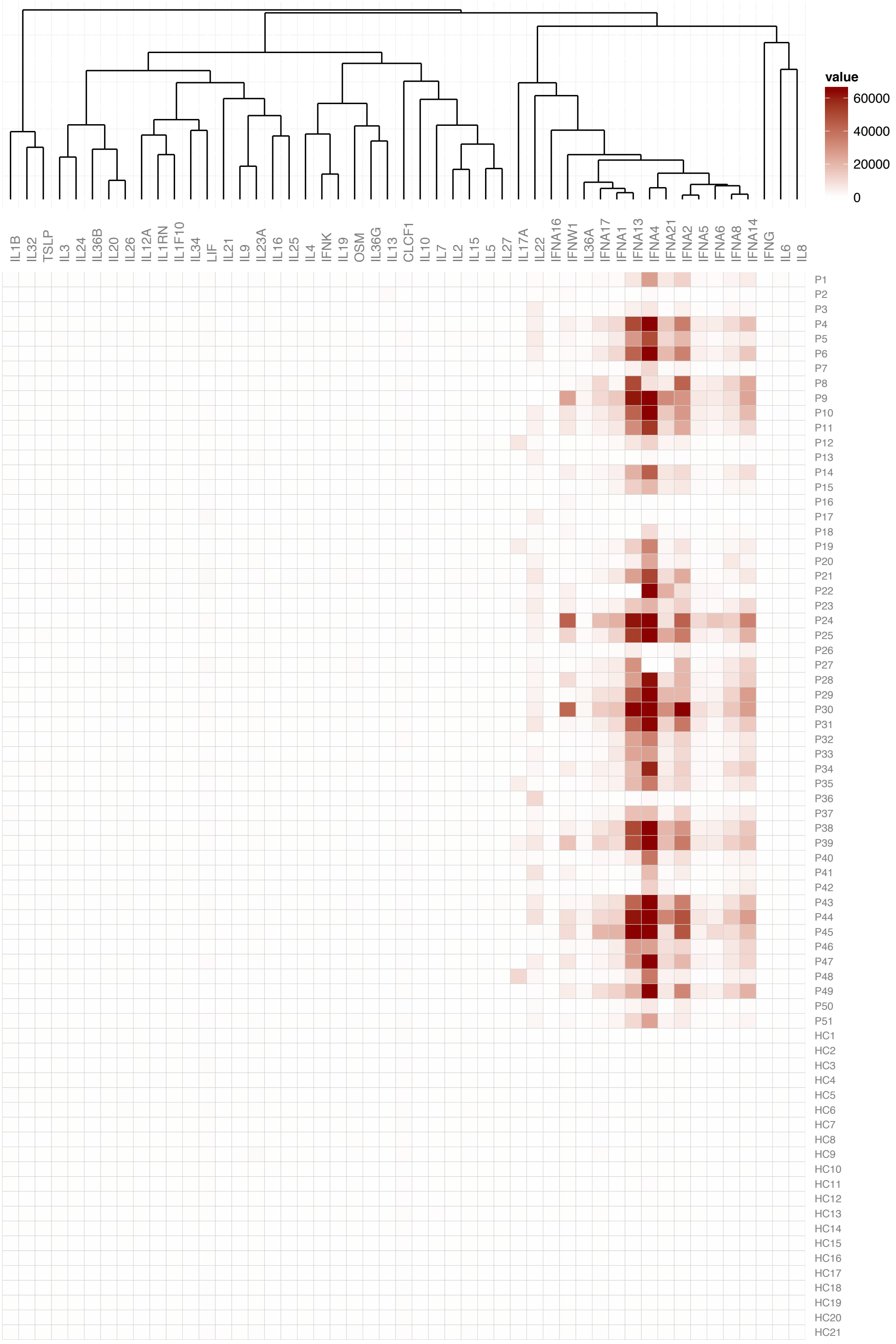

Fig. S7. Focused investigation of 49 interferons, interleukins and other closely related cytokines. High autoantibody signals were seen specifically for the previously

established immune targets, including multiple alpha interferon specifies, IFNW1, IL22 and IL17A (IL17F was not present in the array panel)

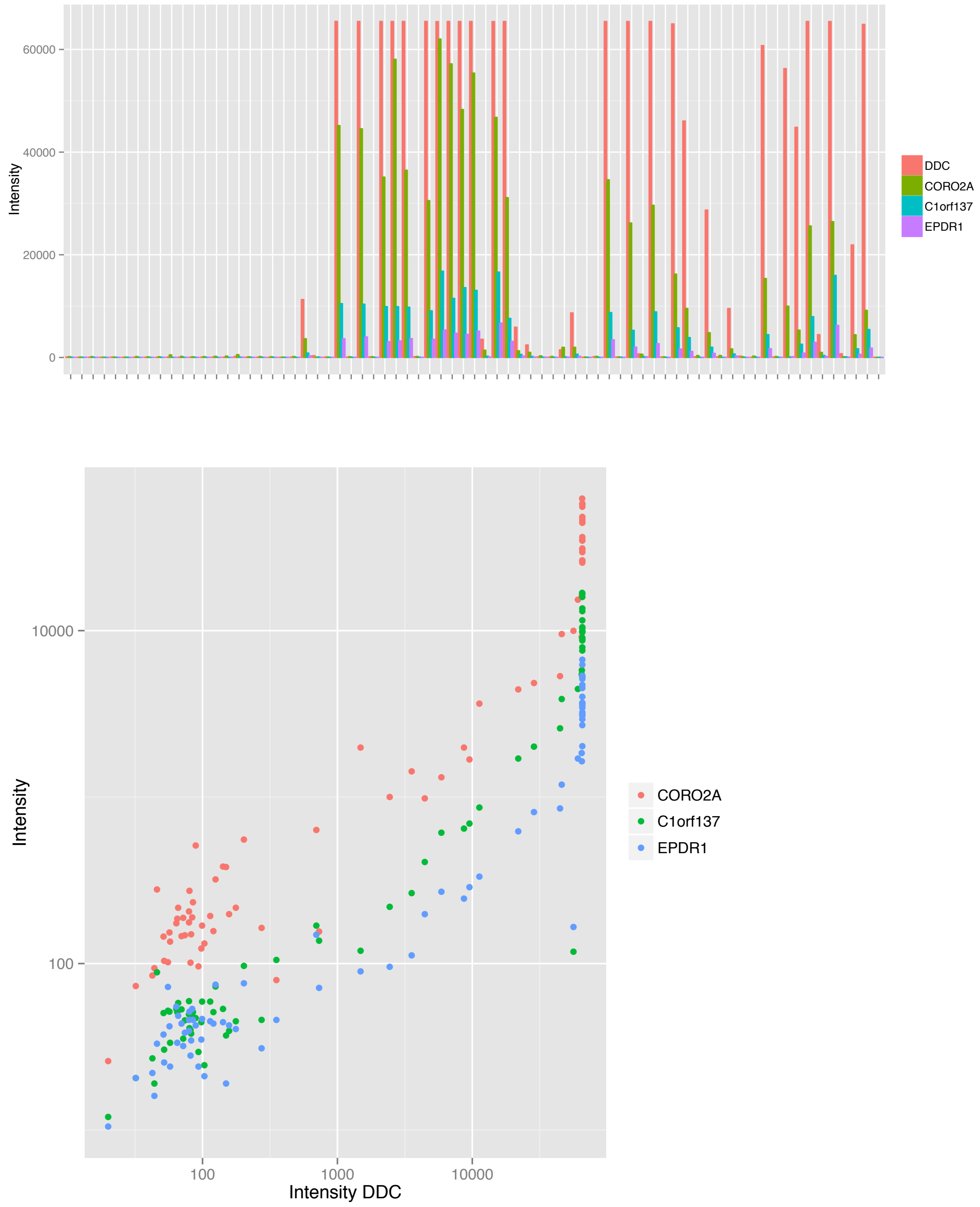

Fig. S8. Printing contamination artifacts exemplified by DDC and adjacent targets. Correlated protein array signals between DCC, an established APS1 autoantigen, and

nearby located proteins; CORO2AA, C1orf137 and EPDR1. Healthy subjects (n=21) are grouped to the left and patients with APS1 (n=51) to the right in the upper image. Average fluorescence signal intensities for protein duplicates on the array after subtraction of the background signal are shown.

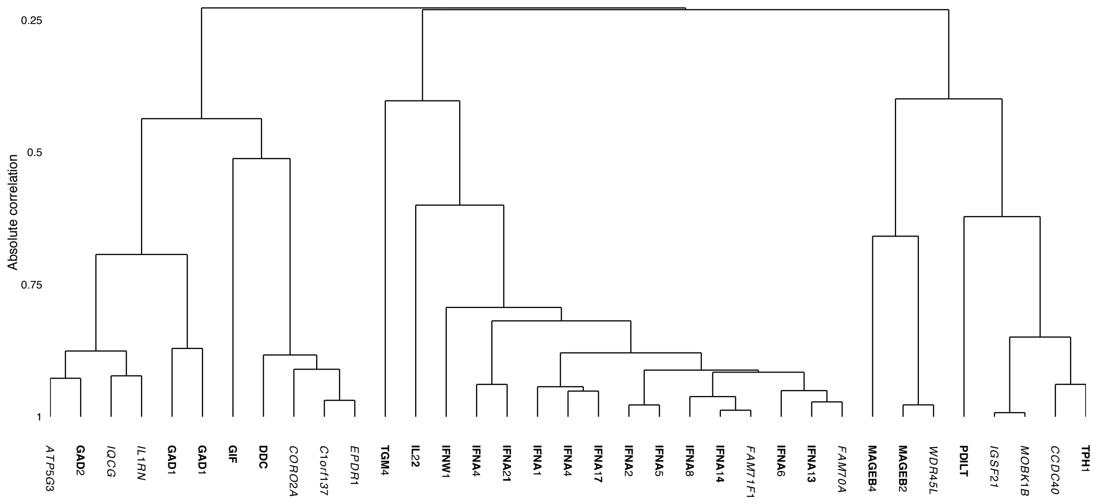

Fig. S9. Identification of printing contamination artifacts in the proteome array data set. Correlations between the top targets in the protein array screen before annotation are shown. Unexpected strong correlations between targets closely located on the array were identified as artifacts (marked in Italic) and excluded from the analyses.

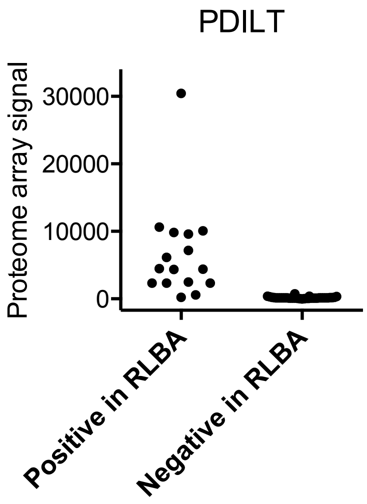

Fig. S10. Validation of PDILT autoantibodies in the APS1 discovery cohort (n=51) using a radio-ligand binding assay (RLBA). Patients that were positive for PDILT autoantibodies in the RLBA are grouped to the left. The cut-off in the RLBA was defined as an index-value of 15. Proteome array signals are shown as the average fluorescence signal intensities for protein duplicates on the array after subtraction of the background signal.

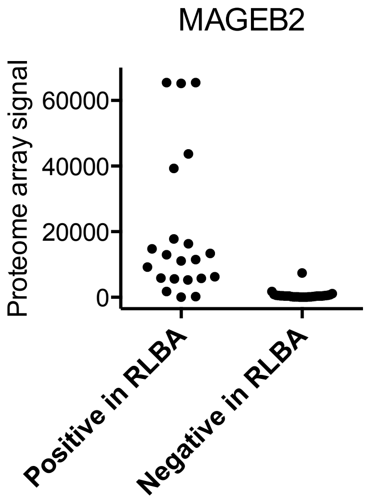

Fig. S11. Validation of MAGEB2 autoantibodies in the APS1 discovery cohort (n=51) using a radio-ligand binding assay (RLBA). Patients that were positive for MAGEB2

autoantibodies in the RLBA are grouped to the left. The cut-off in the RLBA was defined as an index-value of 15. Proteome array signals are shown as the average fluorescence signal intensities for protein duplicates on the array after subtraction of the background signal.

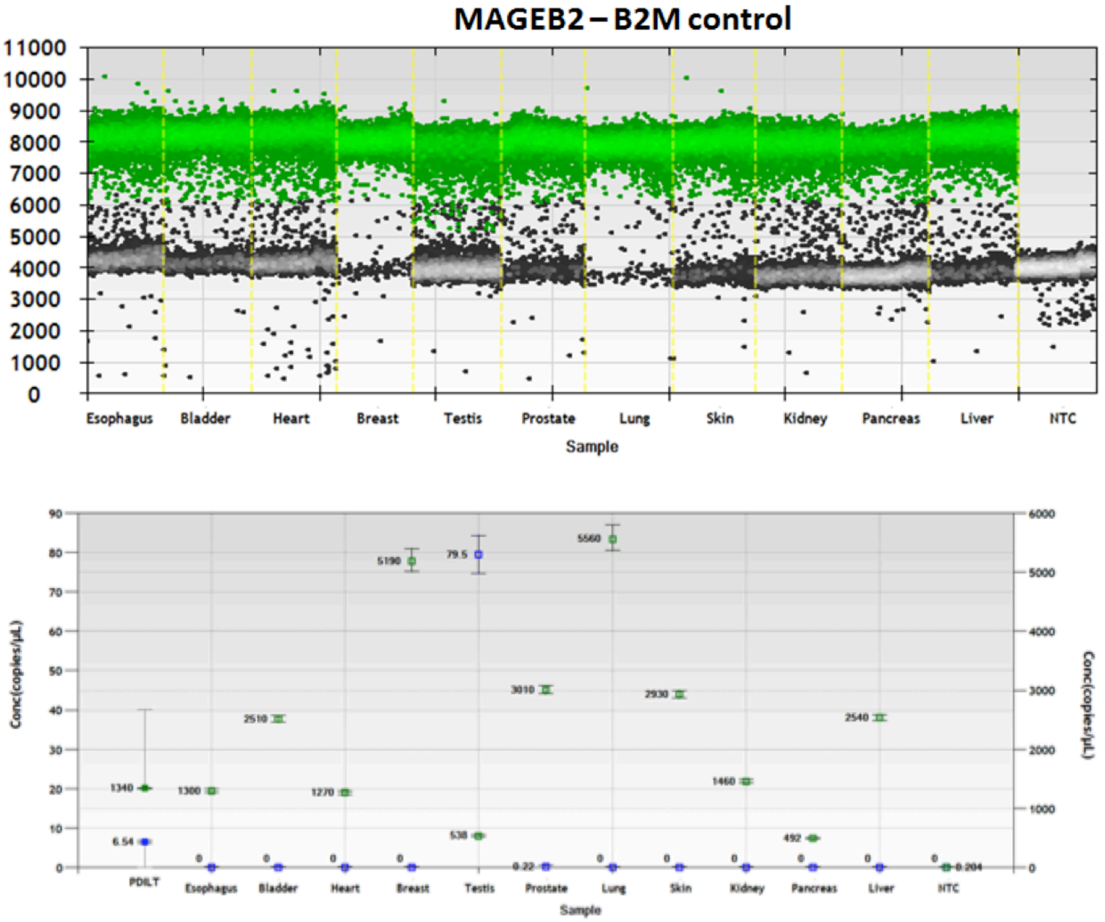

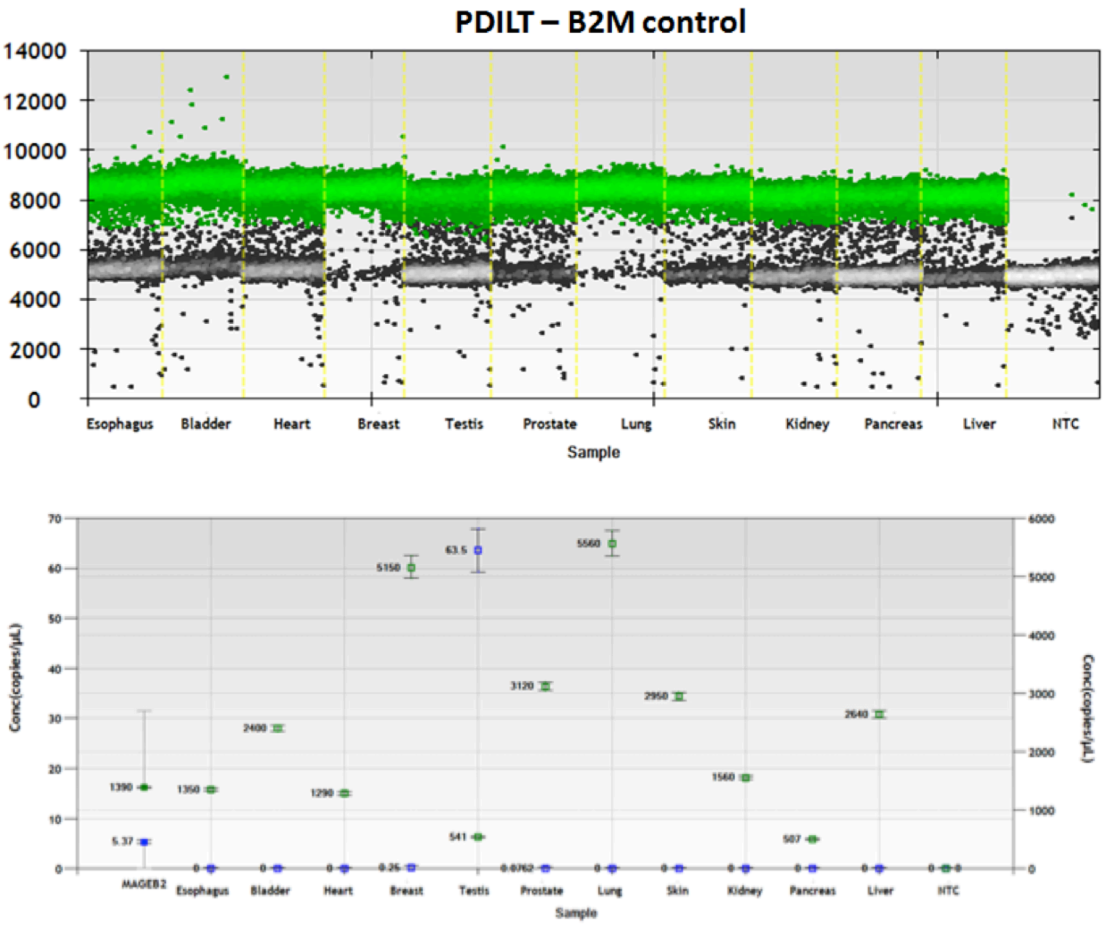

Fig. S12. mRNA levels of MAGEB2 and PDILT were assessed in multiple human tissues by digital droplet PCR. The greatest number of high amplitude events is seen in the testis cDNA sample, and very few or no high amplitude events are seen for the other tissue samples and the *no template control* (NTC). Grey dots denote events with intensities below cut-off. The results for PDILT and MAGEB2 were compared against that of β2M.

Autoantigen	Prevalence	
**IFNW1**	47/51	92%
**IFNA1**	46/51	90%
**DDC**	37/51	73%
**IL22**	35/51	67%
**TPH1**	28/51	55%
**GAD2**	22/51	43%
**GAD1**	15/51	29%
**GIF**	14/51	27%
**IL17A**	12/51	24%
**KCNRG**	3/51	6%
**CYP1A2**	2/51	4%
**TSGA10**	2/51	4%

Table S1. Prevalence of established autoantibodies in the protein array screen. 51 patients with APS1 were investigated. The upper limit of the normal range was defined as three standard deviations above the average of the healthy control subjects.

Gene	Tissue expression, mRNA seq (HPA)
**TPH1**	Group enriched (colon, duodenum, rectum, small intestine, stomach).
**DDC**	Tissue enhanced (duodenum, kidney, small intestine).
**GAD2**	Tissue enriched (cerebral cortex).
**GAD1**	Tissue enriched (cerebral cortex).
**IFNA13**	Not detected.
**IL22**	Not detected.
**GIF**	Tissue enriched (stomach).
**MAGEB2**	Tissue enriched (testis).
**MAGEB4**	Tissue enriched (testis).
**PDILT**	Group enriched (stomach, testis).
**TGM4**	Tissue enriched (prostate).

Table S2. Tissue expression profiles of the major immune targets in the proteome array screen. mRNA sequencing data on gene expression profiles obtained from the Human Protein Atlas, http://www.proteinatlas.org. For comparison, among 20,344 genes investigated by the Human Protein Atlas, 2,355 (12%) were classified as being *Tissue enriched*, 1,109 as *Group enriched* (5%), 3,478 (17%) as *Tissue enhanced*, 8,874 (44%) as *Expressed in all*, 2,696 (13%) as *Mixed* and 1,832 (9%) as *Not detected* [39]. Several type 1 interferon species were major immune targets in the patients with APS1 but are here represented by IFNA13 only.

Best GOs	GO description	Genes	Group count	Total count	P-value (Benjamini corrected)
**GO:0042136**	neurotransmitter biosynthetic process	TPH1 GAD2 GAD1	3	16	1.05e-05
**GO:0006540**	glutamate decarboxylation to succinate	GAD2 GAD1	2	2	1.23e-05
**GO:0006105**	succinate metabolic process	GAD2 GAD1	2	2	1.23e-05
**GO:0004351**	glutamate decarboxylase activity	GAD2 GAD1	2	2	1.23e-05
**GO:0042133**	neurotransmitter metabolic process	TPH1 GAD2 GAD1	3	32	1.86e-05
**GO:0006538**	glutamate catabolic process	GAD2 GAD1	2	3	2.47e-05
**GO:0016831**	carboxy-lyase activity	GAD2 DDC GAD1	3	56	7.37e-05
**GO:0005615**	extracellular space	IFNW1 IL22 IL17A GIF IFNA13	5	528	7.96e-05
**GO:0006519**	amino acid and derivate metabolic process	IL22 TPH1 GAD2 DDC GAD1	5	548	8.48e-05
**GO:0019842**	vitamin binding	GAD2 GIF DDC GAD1	4	245	9.17e-05
**GO:0001505**	regulation of neurotransmitter levels	TPH1 GAD2	3	78	0.000117

		GAD1			
**GO:0009308**	amine metabolic process	IL22<br>TPH1<br>GAD2<br>DDC<br>GAD1	5	636	0.000117
**GO:0007267**	cell-cell signaling	IL22<br>IL17A<br>TPH1<br>GAD2<br>GAD1	5	640	0.000117
**GO:0016830**	carbon-carbon lyase activity	GAD2<br>DDC<br>GAD1	3	82	0.000117
**GO:0005132**	interferon-alpha/beta receptor binding	IFNW1<br>IFNA13	2	9	0.000118
**GO:0006807**	nitrogen compound metabolic process	IL22<br>TPH1<br>GAD2<br>DDC<br>GAD1	5	678	0.000134
**GO:0005125**	cytokine activity	IFNW1<br>IL22<br>IL17A<br>IFNA13	4	310	0.000137
**GO:0019752**	carboxylic acid metabolic process	IL22<br>TPH1<br>GAD2<br>DDC<br>GAD1	5	795	0.000238
**GO:0006082**	organic acid metabolic process	IL22<br>TPH1<br>GAD2<br>DDC<br>GAD1	5	798	0.000238
**GO:0030170**	pyridoxal phosphate binding	GAD2<br>DDC<br>GAD1	3	117	0.000238

**GO:0043648**	dicarboxylic acid metabolic process	GAD2<br>GAD1	2	16	0.000268
**GO:0006536**	glutamate metabolic process	GAD2<br>GAD1	2	16	0.000268
**GO:0044421**	extracellular region part	IFNW1<br>IL22<br>IL17A<br>GIF<br>IFNA13	5	910	0.000386
**GO:0009065**	glutamine familty amino acid catabolic process	GAD2<br>GAD1	2	20	0.000389
**GO:0006520**	amino acid metabolic process	IL22<br>TPH1<br>GAD2<br>GAD1	4	474	0.00049
**GO:0006100**	tricarboxylic acid cycle intermediate metabolic process	GAD2<br>GAD1	2	32	0.000934
**GO:0042401**	biogenic amine biosynthetic process	TPH1<br>DDC	2	34	0.000982
**GO:0006952**	defense response	IFNW1<br>IL22<br>IL17A<br>IFNA13	4	584	0.000982
**GO:0042398**	amino acid derivate biosynthetic process	TPH1<br>DDC	2	42	0.00145
**GO:0016829**	lyase activity	GAD2<br>DDC<br>GAD1	3	278	0.00207

Table S3. Gene ontology terms showing significant overepresentation in established APS1 autoantigens present on the protein array (TPH1, DDC, GAD2, GAD1, IFNA13, IL22, GIF, MAGEB2, MAGEB4, PDILT, TGM4, IFNW1, IL17A, KCNRG, CYP1A2, TSGA10). Associated GO terms were retrieved from the GOa_human database. Minimal length of considered GO paths was set to 3 (default), and top 30 most significant were

reported. 'False discovery rate' according to Benjamini was used for correcting p-values for multiple testing. Results were not clustered.
